# Potential Regulatory Networks and Heterosis for Flavonoid and Terpenoid Contents in Pak Choi: Metabolomic and Transcriptome Analyses

**DOI:** 10.3390/ijms25073587

**Published:** 2024-03-22

**Authors:** Haibin Wang, Tiantian Han, Aimei Bai, Huanhuan Xu, Jianjun Wang, Xilin Hou, Ying Li

**Affiliations:** 1 State Key Laboratory of Crop Genetics & Germplasm Enhancement and Utilization, College of Horticulture, Nanjing Agricultural University, Nanjing 210095, China; 2020204029@stu.njau.edu.cn (H.W.); 2022104050@stu.njau.edu.cn (T.H.); 2021204026@stu.njau.edu.cn (A.B.); 2022204057@stu.njau.edu.cn (H.X.); wangjianjun@njau.edu.cn (J.W.); hxl@njau.edu.cn (X.H.); 2 Nanjing Suman Plasma Engineering Research Institute, Nanjing Agricultural University, Nanjing 210095, China

**Keywords:** Pak choi, transcriptome analysis, metabolome, flavonoids, terpenoids, heterosis

## Abstract

Pak choi exhibits a diverse color range and serves as a rich source of flavonoids and terpenoids. However, the mechanisms underlying the heterosis and coordinated regulation of these compounds—particularly isorhamnetin—remain unclear. This study involved three hybrid combinations and the detection of 528 metabolites from all combinations, including 26 flavonoids and 88 terpenoids, through untargeted metabolomics. Analysis of differential metabolites indicated that the heterosis for the flavonoid and terpenoid contents was parent-dependent, and positive heterosis was observed for isorhamnetin in the two hybrid combinations (SZQ, 002 and HMG, ZMG). Moreover, there was a high transcription level of flavone 3′-O-methyltransferase, which is involved in isorhamnetin biosynthesis. The third group was considered the ideal hybrid combination for investigating the heterosis of flavonoid and terpenoid contents. Transcriptome analysis identified a total of 12,652 DEGs (TPM > 1) in various groups that were used for comparison, and DEGs encoding enzymes involved in various categories, including “carotenoid bio-synthesis” and “anthocyanin biosynthesis”, were enriched in the hybrid combination (SZQ, 002). Moreover, the category of anthocyanin biosynthesis also was enriched in the hybrid combination (HMG, ZMG). The flavonoid pathway demonstrated more differential metabolites than the terpenoid pathway did. The WGCNA demonstrated notable positive correlations between the dark-green modules and many flavonoids and terpenoids. Moreover, there were 23 *ERF* genes in the co-expression network (r ≥ 0.90 and *p* < 0.05). Thus, *ERF* genes may play a significant role in regulating flavonoid and terpenoid biosynthesis. These findings enhance our understanding of the heterosis and coordinated regulation of flavonoid and terpenoid biosynthesis in pak choi, offering insights for genomics-based breeding improvements.

## 1. Introduction

Pak choi is a leafy vegetable with a high economic value and a high concentration of active compounds, such as flavonoids, carotenoids, organic acids, and amino acids [1]. Flavonoids and terpenoids are plant secondary metabolites that play essential roles in many biological processes and plants’ responses to environmental factors. Heterosis occurs in a variety of species and plays an important role in genetic breeding. Since heterosis has been applied to the production of cereal crops, crossbreeding in vegetables has also rapidly advanced. The extent of heterosis can be influenced by the genetic background of the inbred lines, with the individual contributions varying depending on the combinations, environments, and traits involved [2]. The study of the transcriptional regulation mechanisms and heterosis of flavonoids and terpenoids have great theoretical significance and potential application value. However, our understanding of the coordinated regulation of flavonoids and terpenoids and their heterosis remains lacking.

Numerous studies have identified and investigated the regulation of the biosynthesis of flavonoids and terpenoids in plants. Flavonoids, which are plant-derived secondary metabolites, comprise various compounds, such as anthocyanins, catechin derivatives, flavone C-glycosides, flavones, flavanones, flavonols, and isoflavones. These flavonoid compounds characteristically possess a C_6_-C_3_-C_6_ skeletal structure [3]. Terpenoids, including monoterpenes, sesquiterpenes, and triterpenoids, are another important class of plant secondary metabolites. Terpenoids are derived from one of two biosynthetic pathways: the mevalonic acid (MVA) and plastidial 2-C-methylerythritol 4-phosphate (MEP) pathways. Flavonoid and terpenoid metabolism have been studied in many species, including *Cucumis sativus* [4], *Passiflora edulis* [5], *Cryptomeria fortunei* [6], and *Primula veris* [7]. Metabolomic analysis targeting terpenoids has led to the identification of 50 terpenoid metabolites, including 27 triterpenoid saponins and 11 triterpenes [8]. In seven pak choi cultivars, 513 metabolites, including 92 flavonoid compounds, were detected [9]. Proteomic and metabolomic investigations have elucidated tissue-specific and stage-dependent flavonoid accumulation in *Ginkgo biloba* [10], and selenate was found to affect flavonoids in broccoli florets [11]. Isorhamnetin content varied greatly among varieties with different seed coat colors [12]. Temperature primarily regulates plant secondary metabolites and, particularly, the synthesis of flavonoids and terpenoids. Seasonal variations have the greatest influence on photosynthesis pathways, followed by terpenoid and flavonoid biosynthesis pathways [6]. The aroma of hybrid species is influenced by the genetics related to volatile compounds inherited from distinct parent organisms. The scent characteristics of tea plant hybrids were shaped by the pervasive accumulation of hybrid metabolites—notably, volatile terpenes and purine nucleotides [13]. The metabolite class and content are usually related to genetic background. The p1 region on chromosome 1, which acts as a transcription activator for certain parts of the flavonoid pathway, explained 58.0% of the phenotypic variance in maize silks and demonstrated additive gene action [14]. The triterpene biosynthesis pathway was impacted by a whole-genome duplication event during the ecological adaptation or evolutionary process of the *Panax* species [15]. Through functional analyses, it was demonstrated that the sequence variation in *BnA09MYB47a* was responsible for functional disparities between yellow- and black-seeded *B. napus*. Specifically, the black-seed allele *BnA09MYB47a^ZY821^*, as opposed to the yellow-seed allele *BnA09MYB47a^GH06^*, directly triggered the expression of *BnTT18*, thereby enhancing flavonoid biosynthesis [16]. In an intergeneric hybrid between *Brassica rapa* and *Raphanus sativus*, an analysis of all differentially expressed metabolite pathways revealed the top three pathways—ranked by *p*-value—to be “Flavone and flavonol biosynthesis”, “Phenylpropanoid biosynthesis”, and “Isoflavonoid biosynthesis” [17]. However, studies on the heterosis of flavonoid and terpenoid contents remain scarce.

Plant color is primarily determined by the content and diversity of flavonoids and terpenoids. The flavonoid and terpenoid contents in pak choi of different colors vary greatly. Flavonoids and terpenoids have a complex synthetic regulatory network; however, there have been few studies on their synergistic regulation. An examination of transcriptome data related to flavonoid and terpenoid synthesis revealed numerous single-gene candidates that exhibited high expression levels in leaves and/or flowers [5]. Cold stress regulates flavonoid and terpenoid accumulation in plants through phytohormones, transcription processes, functional enzymes, and epigenetics [18]. MYB21 regulates linalool and flavonol biosynthesis in the floral organs and tissues of *Freesia hybrida* [19,20]. The roles of some key transcription factors (MYBs) in regulating flavonoid and carotenoid biosynthesis for tea quality and flavor have been identified [21]. In pak choi, the synergistic regulation of flavonoids and terpenoids remains unknown.

This research investigated the diversity in the levels, heterosis, composition, and regulatory mechanisms of terpenoids and flavonoids in pak choi by integrating transcriptomic and metabolomic analyses. The findings revealed that group 3 was the ideal hybrid combination for the heterosis of flavonoids and terpenoids, with the flavone compound isorhamnetin displaying positive heterosis in the hybrid combinations in groups 2 and 3. *ERF* genes may play a significant role in regulating flavonoid and terpenoid biosynthesis. This study has the potential to enhance our understanding of the regulatory mechanisms of flavonoids and terpenoids in plants, thereby offering a theoretical foundation for the enhancement of the distinctive quality of pak choi.

## 2. Results

### 2.1. Analysis of Metabolites in Pak Choi Leaves

**Figure 1:**
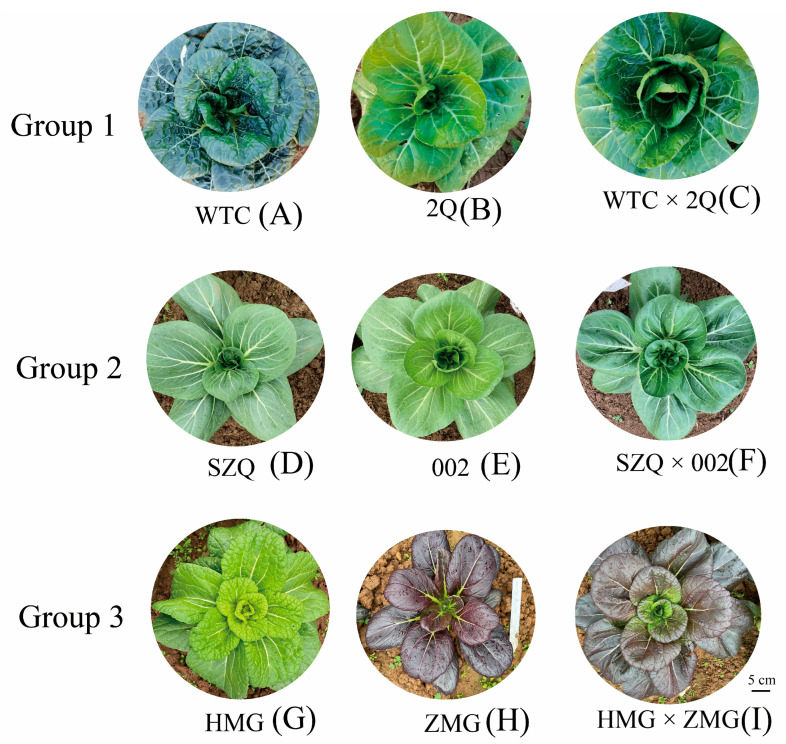
The phenotypes of the parents from the three groups and the hybrid pak choi cultivars used in this study.

**Figure 2:**
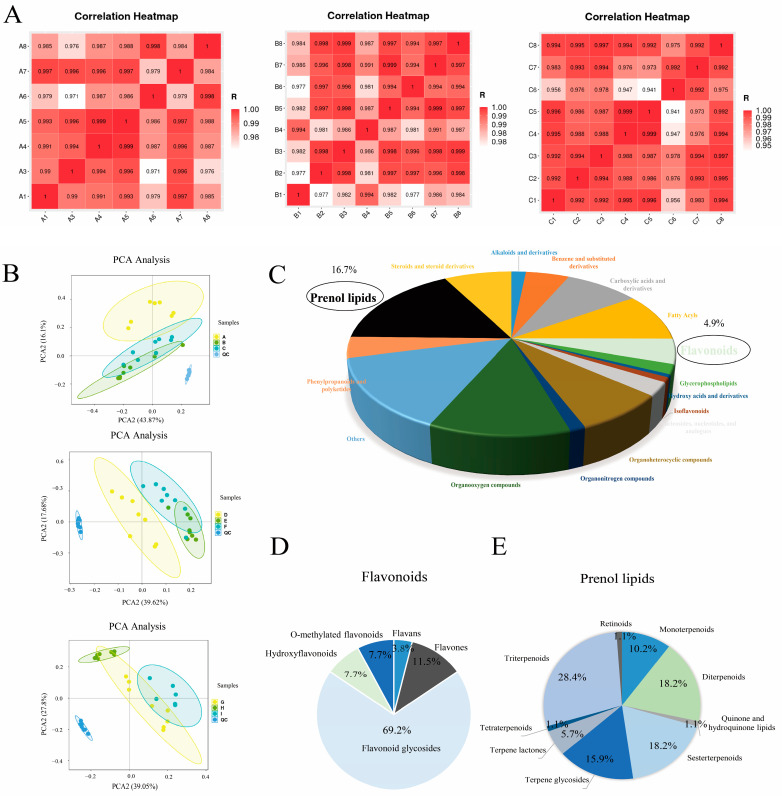
Analysis of metabolites in pak choi leaves. (**A**) Pearson correlation coefficients between replicates of WTC(A), 2Q(B), and WTC × 2Q(C). (**B**) PCA of the differential metabolomes identified in ‘Group 1′, ‘Group 2′, and ‘Group 3′. (**C**) Pie chart depicting the categories of all metabolites. (**D**) Pie chart depicting the categories of all flavonoids. (**E**) Pie chart depicting the categories of all prenol lipids.

The mean height of 2Q was the lowest (10.0 cm), while that of 002 was the highest (19.8 cm). The minimum blade spread was that of SZQ, and the highest was that of HMG (Figure 1) (Table S1). To better understand the types and contents of flavonoid and terpenoid metabolites in NHCC leaves, flavonoid and terpenoid metabolites were analyzed in three groups. Untargeted metabolomic methods were used to identify a total of 528 metabolites in the three groups (Table S2). The eight biological replicates of each sample used for the metabolomic analysis exhibited excellent reproducibility (Figure 2A). In the principal component analysis (PCA), 528 metabolites in different hybrid combinations were classified into three groups according to their PCA1 (43.87%, 39.62.%, and 39.05.%) and PCA2 (16.1%, 27.8%, and 16.1%) values, and each was associated with a characteristic profile (Figure 2B). The identified metabolites were classified into 17 groups, with organic acids, amino acids, quinates and their derivatives, flavonoids, nucleotides and their derivatives, and vitamins being the most concentrated (Table S3). Lipids and flavonoids accounted for 16.7% and 4.9%, respectively (Figure 2C). The classification of flavonoids encompassed seven categories: anthocyanins, catechin derivatives, flavonoids, flavonoid C-glycosides, flavanones, flavonols, and isoflavone compounds. Flavonoid glycosides accounted for 69.2% (Figure 2D). Isoprene lipids primarily included monoterpenes, diterpenes, triterpenes, sesquiterpenes, and terpene glycosides. Triterpenes, sesquiterpenes, and diterpenes accounted for 28.4%, 18.2%, and 18.2%, respectively, making them the top three components in all prenol lipids (Figure 2E). In addition, we calculated the mid-parent heterosis (MPH) of flavonoids and terpenoids. The numbers of terpenoid metabolites with positive heterosis were 2, 6, and 6 in the three groups. The numbers of differential flavonoid metabolites with positive heterosis were 5, 5, and 14 in the three groups. Isomytiloxanthin showed positive mid-parent heterosis in all three hybrid combinations, with the highest rate of heterosis being observed in the third combination (46.43%). Isorhamnetin, a key flavonoid metabolite, exhibited heterosis in groups 2 and 3 (Table S4). Twenty flavonoid and terpenoid metabolites showed mid-parent heterosis, and seventeen metabolites showed higher parent heterosis in the third hybrid combination (Table S4).

### 2.2. Transcriptome Analysis in Pak Choi Leaves

**Figure 3:**
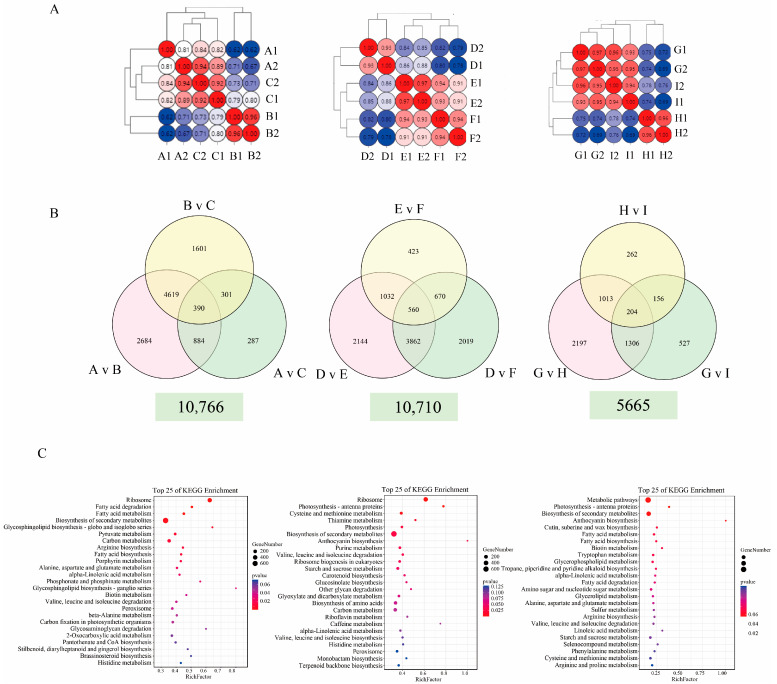
Identification and KEGG analysis of the differentially expressed genes (DEGs). (**A**) Pearson correlation coefficients between transcriptome replicates of A, B, C, D, E, F, G, H, and I. (**B**) Venn diagram of DEGs among the three groups. (**C**) KEGG enrichment analysis of the DEGs in the three groups. The abscissa represents the value of the enrichment factors (the ratio of annotated DEGs to all genes in the enrichment pathways); the ordinate represents the pathways of enrichment. The *q*-value of each term is represented using the color depth. The number of DEGs is represented by the size of the circle.

The changes in gene expression levels among the three hybrid groups were investigated. Nine samples were used as a source of total RNA to construct twenty-seven libraries, followed by RNA-seq profiling. The correlation analysis unveiled a link between F1 and its parental counterparts’ expression patterns (Figure 3A). The phenotype of C (F1 Hybrid) was more closely related to that of parent A. The phenotype of F (F1 Hybrid) was more closely related to that of parent E, and the phenotype of I (F1 Hybrid) was more closely related to that of parent G. For the differential gene expression analysis, we used DESeq2 and selected differentially expressed genes (DEGs) for which |log2FoldChange| > 1 and *Padj*-value < 0.05. A total of 18,510 DEGs were detected in the nine comparison groups of A vs. B, A vs. C, B vs. C, D vs. E, D vs. F, E vs. F, G vs. H, G vs. I, and H vs. I (Table S5). We created a Venn diagram and histogram to better demonstrate the gene expression changes in pak choi leaves in the different hybrid groups. DEGs were observed in three groups: 10,766, 10,710, and 5665 (Figure 3B). To investigate the biological functions of the DEGs (TPM > 1), we analyzed the KEGG pathways, and DEGs encoding enzymes involved in various categories, including “carotenoid biosynthesis” and “anthocyanin biosynthesis”, were enriched in group 2. Moreover, the category of anthocyanin biosynthesis was also enriched in group 3 (Figure 3C). Anthocyanidins play a vital role in mediating pigmentation in plants, and they are an important class of flavonoids that are involved in various physiological reactions.

### 2.3. Association Analysis of Metabolic and Transcriptomic Data

**Figure 4:**
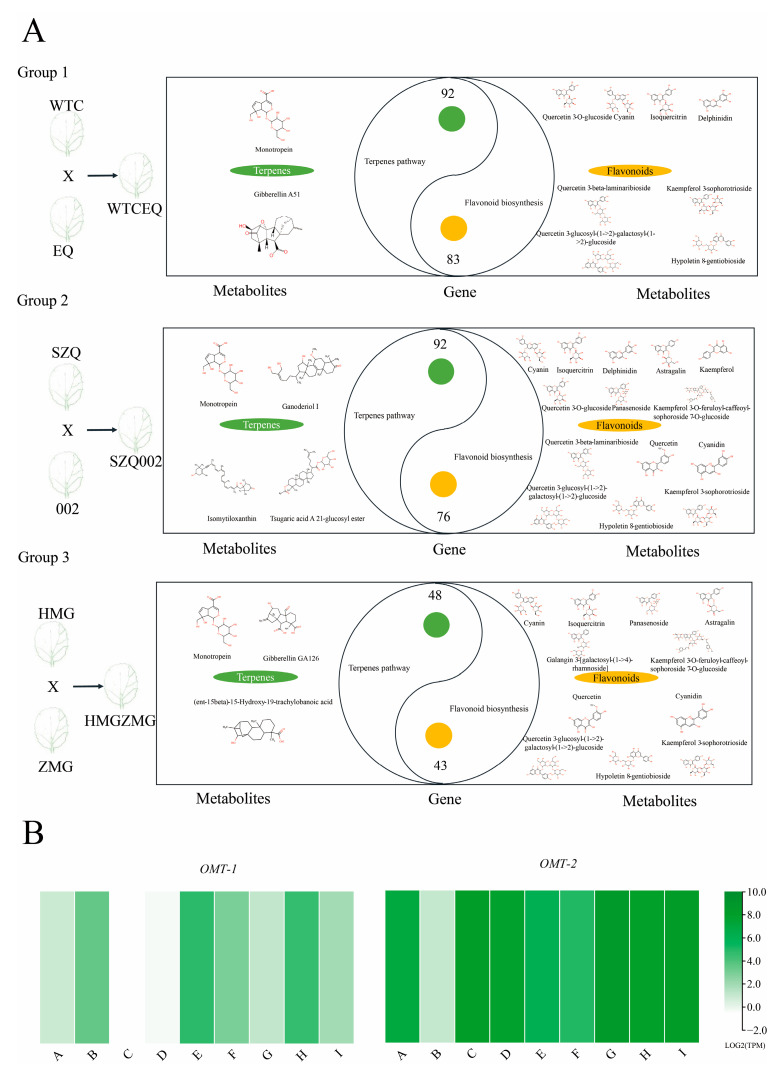
Identification of metabolites and DEGs of terpenes and flavonoids in group 1, group 2, and group 3. (**A**) Total categories of differential metabolites and numbers of differentially expressed genes. (**B**) Transcription levels of key genes for isorhamnetin synthesis. Flavone 3′-O-methyltransferase *OMT-1* (Gene ID: BraC07g015320.1), flavone 3′-O-methyltransferase *OMT-2* (Gene ID: BraC10g012670.1).

In the three groups, 92, 92, and 48 DEGs were mapped to the relevant pathways of “terpene biosynthesis”, including “monoterpenoid biosynthesis”, “sesquiterpenoid and triterpenoid biosynthesis”, “carotenoid biosynthesis”, “brassinosteroid biosynthesis”, “terpenoid backbone biosynthesis”, “zeatin biosynthesis”, and “diterpenoid biosynthesis”, along with two, four, and three differential metabolites. Furthermore, 83, 76, and 43 DEGs were mapped to the relevant pathways of “flavonoid biosynthesis”, including “anthocyanin biosynthesis”, “flavone and flavonol biosynthesis”, “flavonoid biosynthesis”, and “phenylpropanoid biosynthesis”, along with 8, 14, and 11 differential metabolites (Figure 4A). Moreover, flavone 3′-O-methyltransferase (*OMT*), which was involved in isorhamnetin biosynthesis, had a high transcription level (Figure 4B). To further investigate the variations in flavonoid and terpenoid metabolism, a series of tests were conducted using clustering algorithms, and all 528 significant metabolites were divided into 20 clusters based on their accumulation patterns (Figure S1A). According to the analysis of these 20 clusters, Cluster 1 contained many terpenoids. Based on their cumulative patterns, 12,652 DEGs were classified into 20 clusters using a series of tests of clusters (Figure S1B). To analyze the enrichment of genes in cluster 1, KEGG enrichment was used to analyze the DEGs in it (Figure S1C). The results showed that terpenoid backbone biosynthesis was significantly enriched in cluster 1. Based on the results of the series of tests of clusters, it was obvious that the distribution trends of terpenoid-related transcriptomic and metabolomic data in different samples were consistent.

### 2.4. Co-Expression Analysis

**Figure 5:**
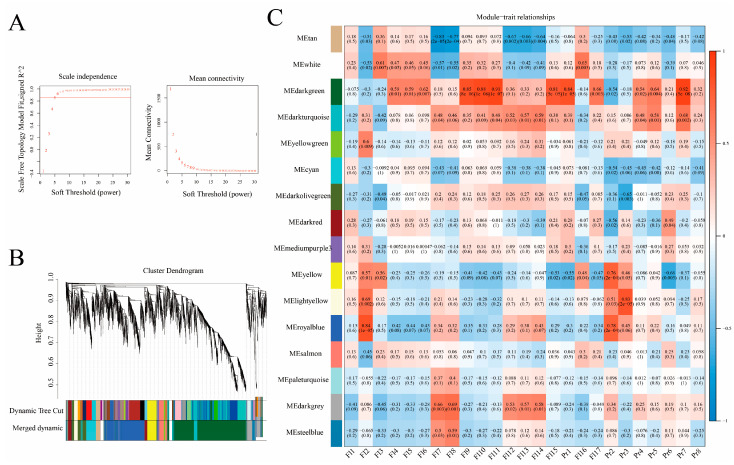
WGCNA results obtained using RNA sequencing data. (**A**) Determination of parameter β of the adjacency function in the WGCNA algorithm. (**B**) Dendrogram showing the hierarchical clustering of the genes, with dissimilarity based on the topological overlap. (**C**) Correlation between modules and phenotypes. Fl1: Quercetin; Fl2: Kaempferol; Fl3: Galangin 3-[galactosyl-(1->4)-rhamnoside]; Fl4: Isoquercitrin; Fl5: Hypoletin 8-gentiobioside; Fl6: Quercetin 3-glucosyl-(1->2)-galactosyl-(1->2)-glucoside; Fl7: Cyanin; Fl8: Kaempferol 3-sophorotrioside; Fl9: Delphinidin; Fl10: Quercetin 3-O-glucoside; Fl11: Quercetin 3-beta-laminaribioside; Fl12: Cyanidin; Fl13: Astragalin; Fl4: Panasenoside; Fl15: Isorhamnetin; Fl16: Kaempferol 3-O-feruloyl-caffeoyl-sophoroside 7-O-glucoside; Fl17: Kaempferol 3-(6-acetylgalactoside); Pr1: Monotropein; Pr2; Gibberellin GA126; Pr3: Tsugaric acid A 21-glucosyl ester; Pr4: 2-Hexaprenyl-3-methyl-6-methoxy-1,4 benzoquinone; Pr5: Ganoderiol I; Pr6: (ent-15beta)-15-Hydroxy-19-trachylobanoic acid; Pr7: Gibberellin A51; Pr8: Isomytiloxanthin.

To further explore the potential co-expression network for flavonoids and terpenoids, a co-expression network based on Pearson correlation coefficients (PCCs) was constructed using 5727 DEGs, including DEGs related to the same cluster of differential flavonoids and terpenoids and 468 differential transcription factors. After determining the optimal parameter (β = 5), the WGCNA algorithm was used to convert the correlation coefficients of gene pairs into adjacent coefficients (Figure 5A,B). WGCNA was then performed to further associate gene expression patterns with the biosynthesis and accumulation of flavonoids and terpenoids. The DEGs were divided into 16 different modules, and genes from the different modules were correlated and measured using Pearson correlation coefficients. The dark-green module had a high correlation (Fl9, Fl10, Fl11, and Fl15 *r* = 0.85, 0.88, 0.91, and 0.81; Pr1 and Pr7, *r* = 0.84 and 0.92) (Figure 5C). Therefore, the dark-green module was considered for the prediction of the co-expression network of flavonoids and terpenoids in pak choi.

### 2.5. Flavonoid and Terpenoid Regulation Networks in Pak Choi Leaves

**Figure 6:**
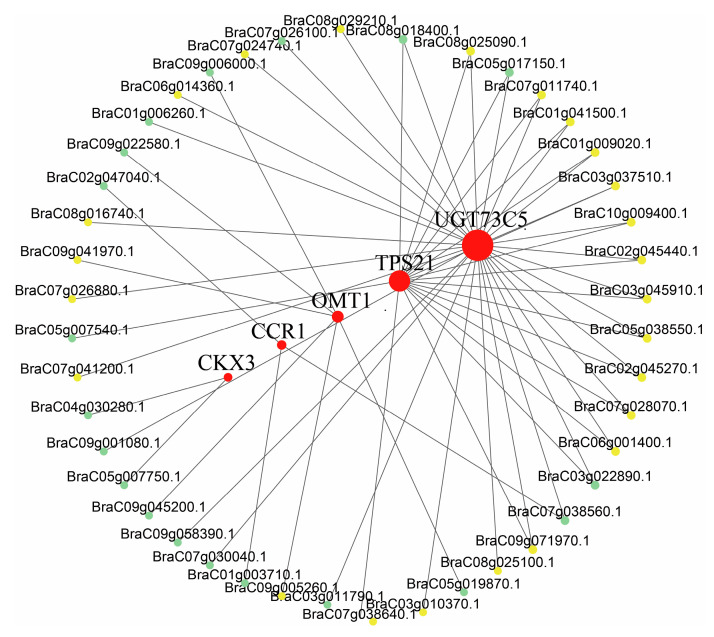
Gene co-expression network of flavonoids and terpenoids in pak choi leaves. The co-expression network analysis in the dark-green module showed the regulatory mechanisms of the key genes involved in terpene and flavonoid biosynthesis with metabolites. Nodes with different colors show terpenoid structural genes, flavonoid structural genes (red), ERF transcription factors (yellow), and other transcription factors (other colors).

Functionally associated genes usually have similar expression patterns because they operate within the same regulatory pathways. To reveal the potential regulation network of flavonoids and terpenoids in pak choi, we analyzed the DEGs related to flavonoids and terpenoids in the dark-green module. This module comprised nine DEGs related to the terpenoid pathway and three DEGs associated with phenylpropanoid biosynthesis. To identify transcription factor (TF) genes contributing to flavonoid and terpenoid accumulation, we selected all DEGs within the dark-green module and assessed the correlations using the Pearson correlation method. We selected DEGs with correlation values of ≥0.90 and *p* < 0.05 to create an interaction network (Figure 6, Table S7) that included five structural genes (*UGT73C5*: BraC03g019410.1; *TPS21*: BraC03g066350.1; *OMT1*: BraC07g027070.1; *CCR1*: BraC09g066370.1; *CKX3*: BraC10g015390.1) and 23 ERF genes. These TFs were potential candidates for regulating the expression of key structural genes in the flavonoid and terpenoid biosynthesis pathways.

### 2.6. Validation of the Key DEGs Involved in Flavonoids and Terpenoids with qRT-PCR

**Figure 7:**
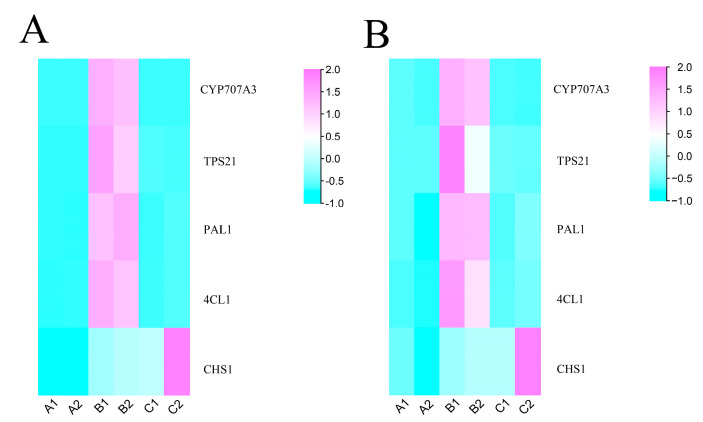
The relative expression levels of genes according to RNA-seq (**A**) and qPCR (**B**).

We randomly selected five flavonoid and terpenoid genes (*PAL1*, *CHS1*, *4CL1*, *TPS21*, *CYP707A3*) for qPCR to detect their transcriptome availability and used the *BcActin* gene as an internal control. The primers for each selected gene were designed using Tbtools (version x64_1_064) (Table S6). The results indicated that the expression patterns of the quantitative data and transcriptome data were consistent (Figure 7), which indicated that the transcriptome analysis was reliable.

## 3. Discussion

Heterosis, which is also known as hybrid vigor, refers to the superior performance of heterozygous hybrids in terms of yield, biomass, and stress tolerance compared to their parental counterparts. Hybridization is a crucial genetic technique in contemporary production practices and plays a vital role in genetic breeding. The selection of parents using breeding techniques is essential for the success of plant breeding programs [22]. In this study, six materials with significant color differences were selected to create hybrid combinations. Heterosis can lead to alterations in the equilibrium between primary and secondary metabolism. For instance, the F1 hybrid plants displayed substantial modifications in primary sugar metabolism compared to their parental plants [23]. The establishment of heterosis in diploid potatoes was primarily attributed to dominant complementation, with hybrids allocating more energy to primary metabolism for rapid growth [24]. However, there is limited research on the heterosis of secondary metabolites—particularly flavonoids and terpenoids. Flavonoids and terpenoids are crucial secondary metabolites that significantly influence the color, taste, and flavor of plants. This study analyzed the heterosis of flavonoids and terpenoids in three hybrid combinations of pak choi by integrating transcriptomic and metabolomic data and establishing gene regulatory networks for differential flavonoids and terpenoids. These findings provide a foundation for breeding high-quality pak choi with valuable secondary metabolites.

Our findings indicate that pak choi is abundant in flavonoids and terpenoids, with flavonoid glycosides being the primary differential flavonoids. Pak choi leaves contain 92 flavonoid compounds, with anthocyanins, flavanone, and flavone-glycosides as the predominant flavonoid groups [9]. We assessed metabolite heterosis using mid-parent heterosis. We identified 13 flavonoid metabolites in group 3 displaying a mid-parent advantage compared to only five in group 1 and group 2. Group 3 is an excellent combination for studying the heterosis of flavonoids. Moreover, terpenoids exhibited greater heterosis in group 2 and group 3. Isorhamnetin, which is known for its anti-obesity effects and functional food properties [25], displayed remarkable positive heterosis in the hybrid combinations in groups 2 and 3. Interestingly, the third combination, which was characterized by the most phenotypic differences, contained the most heterosis of flavonoid and terpenoid metabolites, which was possibly related to genome complementarity [26].

Numerous studies have demonstrated the transcriptional regulation of flavonoid production by specific TFs. In cultivated strawberries, fruit quality and flavonoid biosynthesis are regulated by MYB, bHLH, and WD40 TFs [27]. Interestingly, our potential regulatory network included one TF of phytochrome-interacting factor 4 (*PIF4*) alongside 24 ERF-type TFs and 18 other TFs that may be implicated in flavonoid synthesis. For example, the expression of *PIF4*, along with that of anthocyanin synthase and UDP-glucose:flavonoid 3-o-glucosyl transferase, was enhanced, consequently activating the complete anthocyanin metabolic pathway in red foliated cotton [28]. In regulatory networks, PIF4 plays a pivotal role in the accumulation of anthocyanin in *Arabidopsis*, and this is facilitated by the interactions between PIF4 and PAP1 [29]. Moreover, three AP2/ERF family members modulate flavonoid synthesis by regulating type IV chalcone isomerase in citrus [30] and tomato [31]. ERF can act independently or in conjunction with MYB to regulate flavonoid synthesis; for example, MdERF38 interacts with MdMYB1 in apples [32], while in red pear fruit, the expression of the repressor-type R2R3-MYB gene, *PpMYB140*, is induced by ethylene-activated PpERF105, subsequently inhibiting the biosynthesis of anthocyanin [33]. ERF5 binds to the promoter regions of *MYBA* and *F3H* and transcriptionally activates their gene expression [34]. The AP2/ERF superfamily, a large group of plant-specific TFs, has also been associated with terpenoid regulation. For instance, *PpERF61* overexpression in peach fruit significantly increases linalool content [35]. Transcription factors such as *SlERF.H30* and *SlERF.G6* manipulate carotenoids, a category of terpenoids, by encoding proteins that regulate LYCOPENE-β-CYCLASE 2 (SlLCYB2). SlLCYB2 encodes an enzyme that facilitates the transformation of lycopene into carotene within fruit [36]. In *Salvia miltiorrhiza*, SmERF128 positively regulates diterpenoid biosynthesis [37], and in *Litsea cubeba*, LcERF19 promotes geranial and neral biosynthesis [38]. The presence of ERF in the regulatory network of pak choi implies a potential common regulatory link between flavonoids and terpenoids.

This study presented the first examination of heterosis in flavonoid and terpenoid metabolites in pak choi, revealing variations in different hybrid combinations, particularly those with significant color differences. However, the regulatory network based on positive correlation had limitations in explaining the potential regulatory relationships between transcription factors and structural genes. The findings still offer valuable insights into guiding the breeding of flavonoids and terpenoids.

## 4. Materials and Methods

### 4.1. Plant Material

We selected 3 groups of materials as parents to construct hybrid combinations according to the color and plant type. The pak choi cultivars ‘Wutacai’ (WTC)(A), ‘Erqing’ (2Q)(B), ‘Suzhouqing’ (SZQ)(D), ‘Aijiaohuang’ (002)(E), ‘Huangmeigui’ (HMG)(G), and ‘Zimeigui’ (ZMG)(H) were grown on the Baima farm (Nanjing, China). F1 Hybrids were obtained in April 2020. All samples in this study were sown in September of 2020 and collected for use two months later. The leaves of WTC, EQ, the F1 Hybrid (WTC × 2Q)(C), SZQ, 002, the F1 Hybrid (SZQ × 002)(F), HMG, ZMG, and the F1 Hybrid (HMG × ZMG)(A) were collected and immediately frozen in liquid nitrogen for untargeted metabolomic and transcriptomic analyses (Figure 1). Three biological replicates were set for each treatment.

### 4.2. Metabolite Extraction and Detection

All freeze-dried samples (200 mg) were accurately weighed and added to a centrifuge tube containing 1 mL of 80% ethanol and 100 μM internal standard (Ricinoleic acid). The mixture was immediately ground in a high-throughput tissue crusher (Wonbio-96c; Shanghai Wanbo Biotechnology, Shanghai, China) for 2 min (70 Hz), and the sample was stored at 4 °C for 10 min, followed by centrifugation at 12,000× *g* at 4 °C for 10 min. The supernatant, which was redissolved with methanol, was transferred to a sampling vial with an inner cannula for onboard analysis. Eight biological replicates were assessed for each sample. Metabolite extracts were analyzed on an LC-MS system (UHPLC, Vanquish Horizon UHPLC system; MS, Q-Exactive system; Thermo Fisher Scientific, Waltham, MA, USA). A triple-quadrupole ion trap mass spectrometer (Q TRAP) API 6500 Q TRAP LC/MS/MS system was equipped with an ESI turbo ion-spray interface to conduct linear ion trap (LIT) and triple-quadrupole (QQQ) scanning. The raw data files were imported into the Progenesis QI software (version 3.0, Waters, Manchester, UK) for peak comparison, normalization, peak selection, deconvolution, subsequent compound identification, and statistical analysis. Metabolites were identified by comparing the accurate masses, MS/MS fragmentation patterns, and isotope patterns with those in internal libraries and the Human Metabolome Database (HMDB) (http://www.hmdb.org/).

### 4.3. Transcriptomic Analysis

The total RNA from leaves frozen in liquid nitrogen were extracted and purified using a kit for RNA sequencing and qPCR (TIANGE, Beijing, China). Three biological replicates were performed on each sample. The mappings of clean reads were analyzed and used for HISAT2 v2.1.0 using Tbtools [39]. The number of transcripts per million (TPM) was used for gene-/transcript-level quantification. Differential expression analyses among the three groups were performed with DESeq2. Genes with |log2 fold change (FC)| ≥ 1 and *Padj* < 0.05 were identified as differentially expressed genes (DEGs) and subjected to KEGG enrichment analysis.

### 4.4. Weighted Gene Co-Expression Network Analysis (WGCNA)

The R package (version 4.2.0) of WGCNA was used to analyze the relationship between metabolites and gene expression [40]. Gene expression modules were generated using the TPM of the DEGs, and 25 flavonoid and terpenoid compounds related to metabolite content were used as trait parameters. The correlation coefficients and *p* values were used to express module trait associations. Structural genes related to flavonoid and terpenoid synthesis were selected from the most relevant modules. A regulatory network diagram was drawn using Cytoscape 3.9.1 [41].

### 4.5. Quantitative Real-Time PCR (qPCR) Expression Analyses

The relative expression levels of some flavonoid and terpenoid pathway enzyme genes, including BraC02g034060.1, BraC03g066350.1, BraC04g028280.1, BraC05g019190.1, and BraC10g026540.1, were tested in WTC, 2Q, and their F1 leaves. qPCR was performed with SYBR Premix Ex Taq™ (Takara, Dalian, China). The transcript level of *BcACTIN* was used as an internal control. All qPCR primers are listed in Table S5.

### 4.6. Statistical Analysis

The metabolic product data were exported to the EZinfo 3.0 software for multivariate statistical analysis. Differential levels of metabolites were identified using orthogonal least squares discriminant analysis. The main criteria for significant differences between samples were a projected variable importance (VIP) of ≥1, a maximum multiple change of ≥2, and a *p* value of ≤0.05. Heterosis was quantified as mid-parent heterosis (MPH) = [(F1 − MP)/MP] × 100 and higher parent heterosis (HPH) = [(F1 − HP)/HP] × 100.

## 5. Conclusions

The analysis of the metabolomic data demonstrated significant heterosis in terpenoids and flavonoids in group 3 (HMG, ZMG, and F1). Isorhamnetin, a health-promoting compound, exhibited significant positive heterosis in the hybrid combinations in group 2 and group 3. This finding suggests that hybridization can augment the levels of flavonoids and terpenoids, providing a robust basis for the selection of optimal hybrid materials. Furthermore, an integrated examination of metabolomic and transcriptomic data showed that ERF genes may play a significant role in regulating flavonoid and terpenoid biosynthesis. These findings contribute to the elucidation of the molecular mechanisms and regulatory networks of flavonoid and terpenoid biosynthesis in pak choi, providing a biological basis for the improvement of hybrid breeding through genomics.

## Data Availability

Data is contained within the article and Supplementary Materials.

## Supplementary Materials

The following supporting information can be downloaded at: https://www.mdpi.com/article/10.3390/ijms25073587/s1.
