# Supplementary material for: Potential Regulatory Networks and Heterosis for Flavonoid and Terpenoid Contents in Pak Choi: Metabolomic and Transcriptome Analyses
Source: Int J Mol Sci. 2024 Mar 22;25(7):3587. doi: 10.3390/ijms25073587 (PMC11011442; doi:10.3390/ijms25073587)
Supplement: Supplementary file 1 [file ijms-25-03587-s001.zip › Figure S1.pdf]

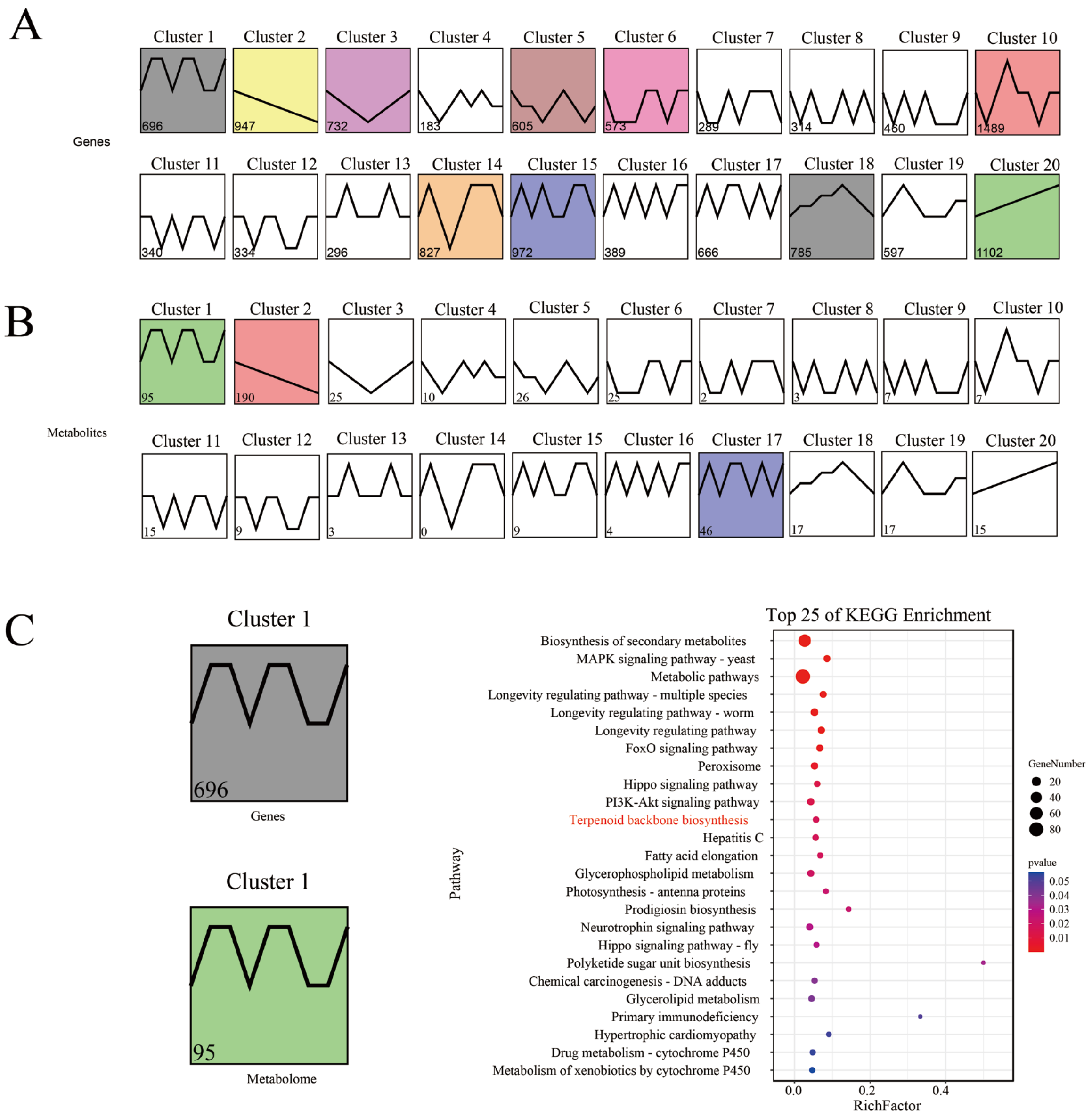

Figure S1. Dynamics of metabolites and gene expression in different materials. (A and B). Series test of cluster grouping pak choi metabolites (A) and transcriptome (B) into twenty clusters. (C). Enlarged view of cluster I in metabolite clustering analysis and cluster 1 in DEGs clustering analysis. KEGG enrichment analysis of cluster 1 in the clustering analysis of DEGs.
